# Crystal structure of sodium (1*S*)-d-lyxit-1-yl­sulfonate

**DOI:** 10.1107/S2056989016005375

**Published:** 2016-04-05

**Authors:** Alan H. Haines, David L. Hughes

**Affiliations:** a School of Chemistry, University of East Anglia, Norwich NR4 7TJ, England

**Keywords:** crystal structure, d-lyxose bis­ulfite adduct, sodium hydrogen sulfite, sodium metabisulfite

## Abstract

The title compound, Na^+^·C_5_H_11_O_8_S^−^ [systematic name: sodium (1*S*,2*S*,3*S*,4*R*)-1,2,3,4,5-penta­hydroxy­pentane-1-sulfonate], is formed by reaction of d-lyxose with sodium bis­ulfite (sodium hydrogen sulfite) in water. The anion has an open-chain structure in which one of the oxygen atoms of the sulfonate residue, the S atom, the C atoms of the sugar chain and the O atom of the hy­droxy­methyl group form an essentially planar zigzag chain with the corresponding torsion angles lying between 179.80 (11) and 167.74 (14)°. A three-dimensional bonding network exists in the crystal structure involving hexa­coordination of sodium ions by O atoms, three of which are provided by a single d-lyxose–sulfonate unit and the other three by two sulfonate groups and one hy­droxy­methyl group, each from separate units of the adduct. Extensive inter­molecular O—H⋯O hydrogen bonding supplements this bonding network.

## Abstract 2

The anion has an open-chain structure in which one of the oxygen atoms of the sulfate residue, the S atom, the C atoms of the sugar chain and the O atom of the hy­droxy­methyl group form an essentially planar zigzag chain. A three-dimensional bonding network exists in the crystal structure involving hexa­coordination of sodium ions by O atoms, three of which are provided by a single d-lyxose–sulfonate unit and the other three by two sulfonate groups and one hy­droxy­methyl group, each from separate units of the adduct. Extensive inter­molecular O—H⋯O hydrogen bonding supplements this bonding network.

## Chemical context   

**Figure 1:**
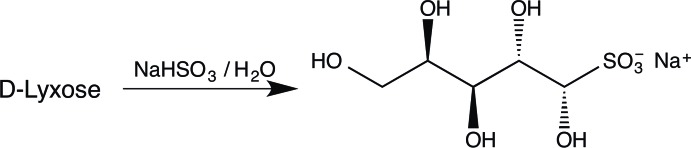
Schematic representation of the preparation of the title compound.

Bisulfite adducts of aldehydes are important compounds because, in many cases, they are crystalline and allow a means of purification and storage of those aldehydes which are liquids or which suffer from problems of instability. The importance of aldehydes in many synthetic processes for the production of commercially important compounds, including pharmaceuticals, means that there is continuing inter­est in these bis­ulfite adducts. A recent publication (Kissane *et al.*, 2013 ▸) has focused on counter-ion effects in the preparation of aldehyde–bis­ulfite adducts. Of particular concern in that work was a comparison of the physical properties of sodium and potassium bis­ulfite adducts of a range of aldehydes, to include their hygroscopic nature and ease of filtration, in order to facilitate their preparation and storage on a large scale. Studies by X-ray crystallography on the bis­ulfite adducts of common aldehydo-sugars such as d-glucose (Cole *et al.*, 2001 ▸) and our related work on d-galactose (Haines & Hughes, 2010 ▸), d-ribose (Haines & Hughes, 2014 ▸) and d-lyxose (Haines & Hughes, 2015 ▸) indicated the crystallinity and ease of isolation of such potassium adducts, and also, in the case of the sodium bis­ulfite adduct of d-glucose (Haines & Hughes, 2012 ▸), allowed a comparison of the potassium and sodium compounds. We now report the preparation (Fig. 1 ▸), properties, and crystal structure of the sodium bis­ulfite adduct of d-lyxose, which allows a further comparison of the influence of the two counter-ions in the properties of an adduct from the same substrate.
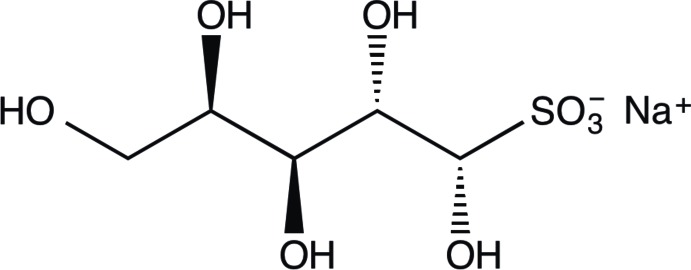


Crystallization of the sodium bis­ulfite adduct of d-lyxose from water required a very concentrated solution from which highly crystalline material grew slowly on storage at room temperature. In contrast to the potassium adduct (Haines & Hughes, 2015 ▸), the crystals lacked water of crystallization but had the same *S*-configuration at C1, leading to a similar positive optical rotation for the two products. The melting points of the sodium and potassium adducts (417.6–420.1 K and 392–400 K, respectively, both with decomposition) were above that of d-lyxose (381–385 K). Both of the d-lyxose adducts were stable on storage in a sealed container at room temperature.

## Structural commentary   

**Figure 2:**
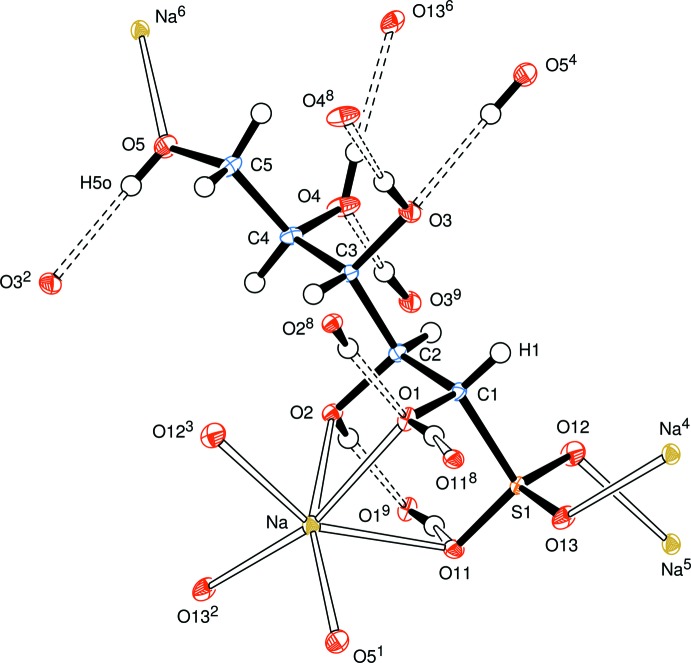
View of the d-lyxose–NaHSO_3_ adduct, indicating the atom-numbering scheme, all sodium coordination contacts and hydrogen bonds involving the atoms of the basic adduct moieties. Displacement ellipsoids are drawn at the 50% probability level. [Symmetry codes: (1) *x*, *y*, *z* − 1; (2) *x*, *y* + 1, *z*; (3) *x* − 1, *y* + 1, *z*; (4) *x*, *y* − 1, *z*; (5) *x* + 1, *y* − 1, *z*; (6) *x*, *y*, *z* + 1; (7) *x* − 1, *y*, *z* + 1; (8) *x* − 1, *y*, *z*; (9) *x* + 1, *y*, *z*.]

**Figure 3:**
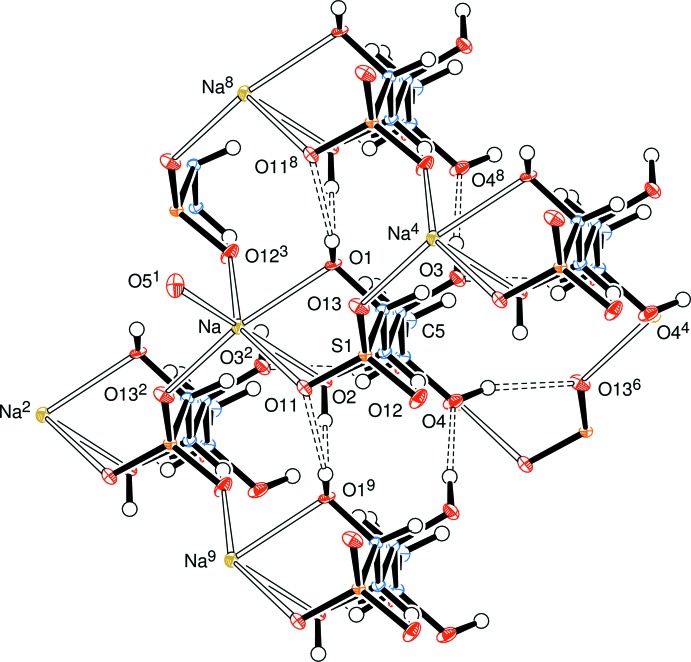
View approximately along the d-lyxose chain, showing the inter­molecular hydrogen bonding and coordination links. Symmetry codes are as in Fig. 2 ▸.

**Figure 4:**
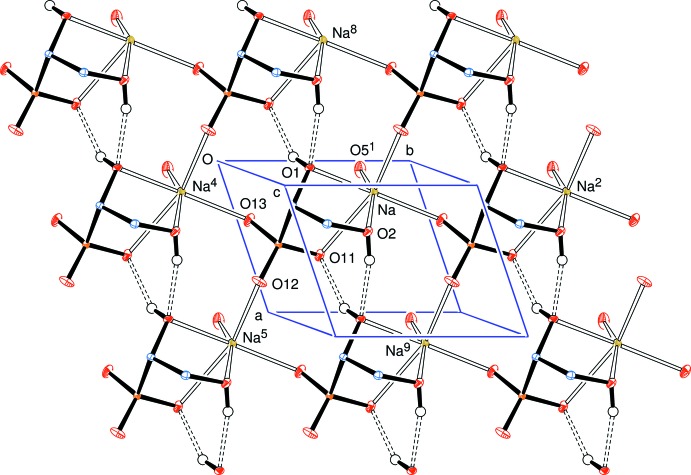
View approximately onto the *ab* plane, showing the links between the sodium ions parallel to that plane. Symmetry codes are as in Fig. 2 ▸.

The newly formed chiral centre at C1 has the *S*-configuration (Fig. 2 ▸) and the systematic name for the salt is sodium (1*S*,2*S*,3*S*,4*R*)-1,2,3,4,5-penta­hydroxy­pentane-1-sulfonate. The anion has an open-chain structure in which one of the oxygen atoms, O13, of the sulfonate residue, the S atom, the C atoms of the sugar chain and the oxygen atom, O5, of the terminal hy­droxy­methyl group form an essentially planar zigzag (all-*trans*) chain with the corresponding torsion angles lying between the absolute values of 179.80 (11) and 167.74 (14)°. The atoms O13–C4 form a plane, with C5 and O5 displaced 0.229 (3) and 0.525 (2) Å, respectively, from that mean plane. All of the hydroxyl groups form medium-strength to weak inter­molecular hydrogen bonds which connect mol­ecules in an extensive three-dimensional network (Fig. 3 ▸ and Table 1 ▸). This network is enhanced through complexation of the sodium atom which has a coordination sphere of six oxygen atoms with an approximately octa­hedral pattern in which three sites are occupied by oxygen atoms O1, O2, and O11 of one basic d-lyxose-sulfonate unit and the remaining three sites are occupied by oxygen atoms O12 and O13 arising from two different sulfonate groups, and O5 of another d-lyxose-sulfonate unit. The Na—O bond lengths lie in the range 2.2524 (16) to 2.5265 (16) Å. The sodium atoms are linked in planes parallel to the *ab* plane through coordinating sulfonate groups supported by H–O⋯Na coordination and hydrogen bonds (Fig. 4 ▸). There is no symmetry in this space group; all the mol­ecules lie parallel and are arranged by translation parallel to the cell axes.

A comparison of the crystal structures of the sodium and potassium bis­ulfite adducts of d-lyxose illustrates the different coordination requirements of the two alkali metal cations. In the potassium salt hydrate (Haines & Hughes, 2015 ▸), two distinct environments for the cation are observed, involving both hexa- and octa-coordination of oxygen atoms, with each cation lying on a twofold symmetry axis. Oxygen atoms from the water of crystallization provide two of the coordination sites for the octa-coordinate potassium ion. In contrast, the sodium salt lacks water of crystallization and possesses a much simpler crystal structure having one environment only for the cation with hexa-coordination of oxygen atoms. However, in both cases the structures accommodate a nearly planar zigzag chain incorporating the sulfur atom, the five sugar carbon atoms and the oxygen of the terminal hy­droxy­methyl group, and both adducts crystallize with the same *S*-configuration at the newly formed chiral centre, despite evidence for the existence of the *R*-stereoisomer in solution.

## Supra­molecular features   

A three-dimensional bonding network exists in the crystal structure through (i) hexa-coordination of a sodium cation with oxygens from four different lyxose bis­ulfite residues, three of those oxygens coming from one such residue, and (ii) inter­molecular hydrogen bonds from each of the five hydroxyl groups to acceptor oxygens in four different residues.

## Spectroscopic findings   

High resolution mass spectrometry in negative ion mode showed no peak for ([C_5_H_11_O_8_S_1_]^−^) at *m*/*z* 231.0108 but a significant peak was observed at 213.0075 ([C_5_H_11_O_8_S_1_– H_2_O]^−^). The mono-anion of d-lyxose gave a peak at *m/z* 149.0458 ([C_5_H_9_O_5_]^−^) and the base peak of the spectrum, observed at *m/z* 299.0982 ([C_10_H_19_O_10_]^−^), was assigned to a dimer ion ([2*M* – H] ^−^) produced by association of a d-lyxose mol­ecule (*M* = C_5_H_10_O_5_) with the mono-anion of d-lyxose ([C_5_H_9_O_5_]^−^) under the electrospray ionization conditions of the mass spectrometric measurement.

The ^1^H NMR spectrum of the adduct in D_2_O indicated the presence of the α- and β-pyran­ose forms of d-lyxose and the major and minor forms of the acyclic sulfonate in the % ratios 11.62 : 5.47 : 74.78 : 8.14. Clearly, the *R*-stereoisomer at C1 is present in solution but only the *S*-isomer crystallizes. Further, some hydrolysis of the adduct to afford the parent sugar occurs during the NMR measurement. As expected, the NMR spectrum of the sodium bis­ulfite adduct is very similar to that of the related potassium sulfite adduct reported earlier (Haines & Hughes, 2015 ▸).

The ^13^C NMR spectrum showed signals for C1 nuclei at δ_C_ 94.81, 94.68, 84.21 and 82.17 arising, respectively, from the β- and α-pyran­ose forms of d-lyxose, the minor adduct and the major adduct, in the % ratios of 5.23 : 15.69 : 7.19 : 71.90.

## Synthesis and crystallization   

d-Lyxose (1 g) was dissolved in water (2 ml) and sodium metabisulfite (0.633 g) was added, Fig. 1 ▸. Complete solution was achieved on warming (to *ca* 313 K). Crystallization did not occur on prolonged standing, so the solution was evaporated at *ca* 303 K until the volume was *ca* 1 ml. On further storage, crystals (0.313 g) were deposited, m.p. 417.6–420.1 K (decomp.) and after concentration of the mother liquors, a further crop (0.204 g) was obtained, m.p. 414–420 K; [α]_D_^21^ +8.9 (12 min.) (*c*, 0.68 in 9 : 1 H_2_O : HOAc). ^1^H NMR (D_2_O, 400 MHz, reference *Me*_3_COH at δ_H_ 1.24): δ_H_ 5.00 (*d*, *J*_1,2_ = 4.6 Hz, H-1 of α-pyran­ose), 4.86 (*d*, *J*_1,2_ = 1.4 Hz, H-1 of β-pyran­ose); signals for the major acyclic sulfonate: δ_H_ 4.71 (*d*, *J*_1,2_ = 0.6 Hz, H-1), 4.19 (*dd*, *J*_2,3_ = 9.5 Hz, H-2), 3.99 (*td*, *J*_3,4_ = 6.4, *J*_4,5b_ = 6.4, *J*_4,5a_ =1.2 Hz, H-4), 3.63 (*dd*, *J*_5a,5b_ = − 9.4 Hz, H-5a); for the minor acyclic sulfonate: δ_H_ 4.63 (*d*, *J*_1,2_ = 5.4 Hz, H-1); ratio of major to minor sulfonate = 9.2 : 1. ^13^C NMR (D_2_O, 100 MHz, reference *Me_3_*COH at δ_C_ 30.29): δ_C_ 94.81 (C1, β-pyran­ose), 94.68 (C1, α-pyran­ose); signals for the major acyclic sulfonate: δ_C_ 82.17 (C1), 70.43, 69.85, 69.32 (C2, C3, C4), 63.78 (C5); the minor acyclic sulfonate showed a peak at δ_C_ 84.21 (C1).

Integration of the various signals for H-1 in the ^1^H NMR spectrum indicated the species α-pyran­ose, β-pyran­ose, major acyclic sulfonate and minor acyclic sulfonate were present in the % ratios of 11.62 : 5.47 : 74.78 : 8.14. In the ^13^C NMR spectrum, based on peak heights, the corresponding ratios were: 15.69 : 5.23 : 71.90 : 7.19.

HRESMS (negative ion mode, measured in H_2_O/MeOH, solution) gave a peak at *m*/*z* 149.0458 ([C_5_H_9_O_5_]^−^), a significant peak at 213.0075 ([C_5_H_11_O_8_S_1_ – H_2_O]^−^), and the base peak at 299.0982 ([C_10_H_19_O_10_]^−^). No significant peak was observed for ([C_5_H_11_O_8_S_1_]^−^) at the calculated *m*/*z* of 231.0180.

## Refinement   

Crystal data, data collection and structure refinement details are summarized in Table 2 ▸. Hydrogen atoms were located in difference maps and were refined freely with isotropic displacement parameters, except for H1 and H3 for which the *U*_iso_ values were set at the positive value of 0.010 (rather than refining to a very low or negative value).

## Supplementary Material

Crystal structure: contains datablock(s) I. DOI: 10.1107/S2056989016005375/sj5496sup1.cif

Structure factors: contains datablock(s) I. DOI: 10.1107/S2056989016005375/sj5496Isup2.hkl

Supporting information file. DOI: 10.1107/S2056989016005375/sj5496Isup3.cml

CCDC reference: 1471425

Additional supporting information:  crystallographic information; 3D view; checkCIF report

## Figures and Tables

**Table 1:** Hydrogen-bond geometry (Å, °)

*D*—H⋯*A*	*D*—H	H⋯*A*	*D*⋯*A*	*D*—H⋯*A*
C5—H5*B*⋯O11^i^	0.97 (4)	2.48 (3)	3.394 (2)	157 (3)
O1—H1*O*⋯O11^ii^	0.74 (4)	2.00 (4)	2.6813 (19)	152 (4)
O2—H2*O*⋯O1^iii^	0.88 (4)	1.97 (4)	2.8311 (19)	164 (3)
O3—H3*O*⋯O4^ii^	0.87 (3)	1.79 (3)	2.664 (2)	173 (3)
O4—H4*O*⋯O13^iv^	0.86 (4)	2.11 (4)	2.936 (2)	162 (4)
O5—H5*O*⋯O3^v^	0.80 (5)	1.99 (5)	2.782 (2)	166 (5)

Symmetry codes: (i) 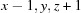; (ii) 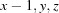; (iii) 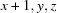; (iv) 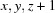; (v) 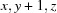.

**Table 2:** Experimental details

Crystal data	
Chemical formula	Na^+^·C_5_H_11_O_8_S^−^
*M*_r_	254.19
Crystal system, space group	Triclinic, *P*1
Temperature (K)	140
*a*, *b*, *c* (Å)	4.8558 (7), 5.8496 (10), 8.7950 (13)
α, β, γ (°)	76.517 (13), 81.528 (12), 71.392 (14)
*V* (Å^3^)	229.51 (7)
*Z*	1
Radiation type	Mo *K*α
μ (mm^−1^)	0.42
Crystal size (mm)	0.37 × 0.22 × 0.15

Data collection	
Diffractometer	Oxford Diffraction Xcalibur 3/Sapphire3 CCD
Absorption correction	Multi-scan (*CrysAlis PRO*; Agilent, 2014 ▸)
*T*_min_, *T*_max_	0.608, 1.000
No. of measured, independent and observed [*I* > 2σ(*I*)] reflections	4256, 2668, 2637
*R*_int_	0.031
(sin θ/λ)_max_ (Å^−1^)	0.703

Refinement	
*R*[*F* ^2^ > 2σ(*F* ^2^)], *wR*(*F* ^2^), *S*	0.023, 0.059, 1.09
No. of reflections	2668
No. of parameters	178
No. of restraints	3
H-atom treatment	H atoms treated by a mixture of independent and constrained refinement
Δρ_max_, Δρ_min_ (e Å^−3^)	0.25, −0.45
Absolute structure	Flack *x* determined using 1289 quotients [(*I* ^+^)−(*I* ^−^)]/[(*I* ^+^)+(*I* ^−^)] (Parsons et al., 2013 ▸)
Absolute structure parameter	0.03 (3)

Computer programs: *CrysAlis PRO* (Agilent, 2014 ▸), *SHELXS97* (Sheldrick, 2008 ▸), *SHELXL2014* (Sheldrick, 2015 ▸), *ORTEPII* (Johnson, 1976 ▸) and *ORTEP-3 for Windows* and *WinGX* (Farrugia, 2012 ▸).

## supplementary crystallographic information

### Crystal data

**Table:**

Na^+^·C_5_H_11_O_8_S^−^	*Z* = 1
*M**_r_* = 254.19	*F*(000) = 132
Triclinic, *P*1	*D*_x_ = 1.839 Mg m^−^^3^
*a* = 4.8558 (7) Å	Mo *K*α radiation, λ = 0.71073 Å
*b* = 5.8496 (10) Å	Cell parameters from 3183 reflections
*c* = 8.7950 (13) Å	θ = 4.0–32.9°
α = 76.517 (13)°	µ = 0.42 mm^−^^1^
β = 81.528 (12)°	*T* = 140 K
γ = 71.392 (14)°	Block, colourless
*V* = 229.51 (7) Å^3^	0.37 × 0.22 × 0.15 mm

### Data collection

**Table:**

Oxford Diffraction Xcalibur 3/Sapphire3 CCD diffractometer	2668 independent reflections
Radiation source: Enhance (Mo) X-ray Source	2637 reflections with *I* > 2σ(*I*)
Graphite monochromator	*R*_int_ = 0.031
Detector resolution: 16.0050 pixels mm^-1^	θ_max_ = 30.0°, θ_min_ = 3.8°
Thin–slice φ and ω scans	*h* = −6→6
Absorption correction: multi-scan (*CrysAlis PRO*; Agilent, 2014)	*k* = −8→8
*T*_min_ = 0.608, *T*_max_ = 1.000	*l* = −12→12
4256 measured reflections	

### Refinement

**Table:**

Refinement on *F*^2^	Secondary atom site location: difference Fourier map
Least-squares matrix: full	Hydrogen site location: difference Fourier map
*R*[*F*^2^ > 2σ(*F*^2^)] = 0.023	H atoms treated by a mixture of independent and constrained refinement
*wR*(*F*^2^) = 0.059	*w* = 1/[σ^2^(*F*_o_^2^) + (0.039*P*)^2^ + 0.0108*P*] where *P* = (*F*_o_^2^ + 2*F*_c_^2^)/3
*S* = 1.09	(Δ/σ)_max_ = 0.001
2668 reflections	Δρ_max_ = 0.25 e Å^−^^3^
178 parameters	Δρ_min_ = −0.45 e Å^−^^3^
3 restraints	Absolute structure: Flack *x* determined using 1289 quotients [(*I*^+^)-(*I*^-^)]/[(*I*^+^)+(*I*^-^)] (Parsons et al., 2013)
Primary atom site location: structure-invariant direct methods	Absolute structure parameter: 0.03 (3)

### Special details

**Table:**

Experimental. Absorption correction: CrysAlisPro (Agilent 2014). Empirical absorption correction using spherical harmonics, implemented in SCALE3 ABSPACK scaling algorithm.
Geometry. All esds (except the esd in the dihedral angle between two l.s. planes) are estimated using the full covariance matrix. The cell esds are taken into account individually in the estimation of esds in distances, angles and torsion angles; correlations between esds in cell parameters are only used when they are defined by crystal symmetry. An approximate (isotropic) treatment of cell esds is used for estimating esds involving l.s. planes.

### Fractional atomic coordinates and isotropic or equivalent isotropic displacement parameters (Å^2^)

**Table:**

	*x*	*y*	*z*	*U*_iso_*/*U*_eq_	
Na	0.14247 (15)	0.62758 (13)	0.42059 (8)	0.00942 (15)	
S1	0.47392 (5)	0.02789 (4)	0.47548 (4)	0.00670 (10)	
O11	0.5597 (3)	0.2447 (2)	0.39414 (15)	0.0105 (2)	
O12	0.7080 (3)	−0.1631 (3)	0.55456 (16)	0.0152 (3)	
O13	0.3305 (3)	−0.0520 (3)	0.37321 (16)	0.0132 (3)	
C1	0.1957 (3)	0.1206 (3)	0.62783 (19)	0.0074 (3)	
C2	0.2966 (4)	0.2336 (3)	0.7408 (2)	0.0080 (3)	
C3	0.0562 (4)	0.3094 (3)	0.86872 (19)	0.0080 (3)	
C4	0.1694 (4)	0.4162 (3)	0.9803 (2)	0.0098 (3)	
C5	−0.0682 (4)	0.5331 (4)	1.0947 (2)	0.0133 (3)	
O1	−0.0431 (3)	0.2976 (2)	0.55444 (15)	0.0090 (2)	
O2	0.3613 (3)	0.4537 (2)	0.66271 (15)	0.0093 (2)	
O3	−0.0376 (3)	0.1035 (3)	0.94619 (16)	0.0123 (3)	
O4	0.4056 (3)	0.2355 (3)	1.05675 (17)	0.0154 (3)	
O5	0.0303 (4)	0.6739 (3)	1.17348 (17)	0.0163 (3)	
H1	0.158 (6)	−0.022 (5)	0.679 (3)	0.010*	
H2	0.450 (6)	0.117 (5)	0.794 (3)	0.010 (6)*	
H3	−0.096 (6)	0.433 (5)	0.816 (3)	0.010*	
H4	0.246 (6)	0.548 (5)	0.919 (3)	0.007 (5)*	
H5A	−0.236 (6)	0.632 (5)	1.037 (4)	0.013 (7)*	
H5B	−0.133 (7)	0.409 (6)	1.171 (4)	0.021 (7)*	
H1O	−0.107 (8)	0.248 (6)	0.503 (4)	0.023 (7)*	
H2O	0.552 (8)	0.420 (7)	0.642 (4)	0.032 (9)*	
H3O	−0.219 (7)	0.158 (6)	0.982 (3)	0.019 (7)*	
H4O	0.345 (9)	0.159 (8)	1.144 (5)	0.034 (9)*	
H5O	0.035 (10)	0.799 (9)	1.113 (6)	0.055 (13)*	

### Atomic displacement parameters (Å^2^)

**Table:**

	*U*^11^	*U*^22^	*U*^33^	*U*^12^	*U*^13^	*U*^23^
Na	0.0114 (3)	0.0081 (3)	0.0094 (3)	−0.0030 (2)	−0.0021 (2)	−0.0021 (2)
S1	0.00646 (16)	0.00679 (16)	0.00738 (16)	−0.00169 (12)	−0.00029 (12)	−0.00294 (11)
O11	0.0130 (6)	0.0106 (6)	0.0095 (5)	−0.0065 (4)	0.0013 (4)	−0.0021 (4)
O12	0.0110 (6)	0.0144 (6)	0.0138 (6)	0.0048 (5)	−0.0017 (5)	−0.0021 (5)
O13	0.0161 (6)	0.0170 (6)	0.0123 (6)	−0.0098 (5)	0.0009 (5)	−0.0083 (5)
C1	0.0065 (7)	0.0075 (7)	0.0082 (7)	−0.0017 (5)	0.0005 (5)	−0.0025 (5)
C2	0.0074 (6)	0.0093 (7)	0.0074 (7)	−0.0020 (5)	−0.0009 (5)	−0.0021 (5)
C3	0.0076 (6)	0.0090 (7)	0.0069 (7)	−0.0020 (6)	−0.0001 (5)	−0.0018 (5)
C4	0.0098 (7)	0.0135 (8)	0.0068 (7)	−0.0042 (6)	−0.0006 (5)	−0.0025 (6)
C5	0.0147 (8)	0.0166 (8)	0.0099 (7)	−0.0043 (6)	0.0009 (6)	−0.0069 (6)
O1	0.0063 (5)	0.0103 (6)	0.0116 (6)	−0.0013 (4)	−0.0036 (4)	−0.0045 (4)
O2	0.0094 (5)	0.0113 (6)	0.0090 (5)	−0.0054 (4)	0.0012 (4)	−0.0035 (4)
O3	0.0115 (6)	0.0111 (6)	0.0139 (6)	−0.0051 (5)	0.0029 (5)	−0.0017 (4)
O4	0.0091 (5)	0.0267 (7)	0.0081 (6)	−0.0025 (5)	−0.0021 (4)	−0.0020 (5)
O5	0.0276 (7)	0.0142 (7)	0.0093 (6)	−0.0067 (6)	−0.0040 (5)	−0.0041 (5)

### Geometric parameters (Å, º)

**Table:**

Na—O5^i^	2.2524 (16)	C1—H1	0.91 (3)
Na—O13^ii^	2.2661 (16)	C2—O2	1.417 (2)
Na—O12^iii^	2.3728 (15)	C2—C3	1.530 (2)
Na—O1	2.3791 (16)	C2—H2	0.93 (3)
Na—O2	2.3800 (15)	C3—O3	1.4136 (19)
Na—O11	2.5265 (16)	C3—C4	1.523 (2)
Na—C1	3.0515 (19)	C3—H3	0.94 (3)
Na—S1	3.3114 (10)	C4—O4	1.417 (2)
Na—S1^ii^	3.3864 (9)	C4—C5	1.512 (2)
Na—S1^iii^	3.3866 (10)	C4—H4	0.98 (3)
S1—O12	1.4435 (14)	C5—O5	1.415 (2)
S1—O13	1.4496 (13)	C5—H5A	0.97 (3)
S1—O11	1.4562 (13)	C5—H5B	0.97 (4)
S1—C1	1.8034 (17)	O1—H1O	0.74 (4)
S1—Na^iv^	3.3865 (9)	O2—H2O	0.88 (4)
S1—Na^v^	3.3866 (10)	O3—H3O	0.87 (3)
O12—Na^v^	2.3728 (15)	O4—H4O	0.86 (4)
O13—Na^iv^	2.2661 (16)	O5—Na^vi^	2.2524 (16)
C1—O1	1.406 (2)	O5—H5O	0.80 (5)
C1—C2	1.523 (2)		
			
O5^i^—Na—O13^ii^	96.36 (6)	O11—S1—Na^iv^	141.71 (5)
O5^i^—Na—O12^iii^	105.63 (6)	C1—S1—Na^iv^	90.25 (6)
O13^ii^—Na—O12^iii^	93.66 (6)	Na—S1—Na^iv^	121.70 (3)
O5^i^—Na—O1	101.70 (6)	O12—S1—Na^v^	35.88 (6)
O13^ii^—Na—O1	161.60 (6)	O13—S1—Na^v^	96.24 (6)
O12^iii^—Na—O1	78.19 (5)	O11—S1—Na^v^	94.58 (6)
O5^i^—Na—O2	161.20 (6)	C1—S1—Na^v^	141.61 (6)
O13^ii^—Na—O2	92.49 (6)	Na—S1—Na^v^	140.21 (3)
O12^iii^—Na—O2	90.27 (5)	Na^iv^—S1—Na^v^	91.60 (2)
O1—Na—O2	71.30 (5)	S1—O11—Na	109.54 (7)
O5^i^—Na—O11	91.52 (6)	S1—O12—Na^v^	123.24 (8)
O13^ii^—Na—O11	107.60 (5)	S1—O13—Na^iv^	130.10 (9)
O12^iii^—Na—O11	151.12 (6)	O1—C1—C2	108.62 (13)
O1—Na—O11	75.63 (5)	O1—C1—S1	107.39 (11)
O2—Na—O11	69.96 (5)	C2—C1—S1	112.68 (11)
O5^i^—Na—C1	115.69 (6)	O1—C1—Na	49.01 (8)
O13^ii^—Na—C1	142.37 (6)	C2—C1—Na	81.73 (9)
O12^iii^—Na—C1	95.93 (5)	S1—C1—Na	81.65 (6)
O1—Na—C1	26.49 (4)	O1—C1—H1	113.8 (17)
O2—Na—C1	51.26 (5)	C2—C1—H1	110.4 (17)
O11—Na—C1	55.35 (5)	S1—C1—H1	104.0 (16)
O5^i^—Na—S1	96.73 (5)	Na—C1—H1	162.5 (17)
O13^ii^—Na—S1	130.25 (5)	O2—C2—C1	111.14 (13)
O12^iii^—Na—S1	127.92 (5)	O2—C2—C3	105.16 (13)
O1—Na—S1	51.16 (3)	C1—C2—C3	111.34 (13)
O2—Na—S1	65.10 (4)	O2—C2—H2	113.6 (17)
O11—Na—S1	24.48 (3)	C1—C2—H2	109.9 (17)
C1—Na—S1	32.60 (3)	C3—C2—H2	105.4 (16)
O5^i^—Na—S1^ii^	115.37 (5)	O3—C3—C4	112.49 (13)
O13^ii^—Na—S1^ii^	19.11 (4)	O3—C3—C2	109.19 (13)
O12^iii^—Na—S1^ii^	89.97 (4)	C4—C3—C2	109.33 (13)
O1—Na—S1^ii^	142.89 (4)	O3—C3—H3	110.7 (18)
O2—Na—S1^ii^	73.74 (4)	C4—C3—H3	109.1 (17)
O11—Na—S1^ii^	103.67 (4)	C2—C3—H3	105.7 (17)
C1—Na—S1^ii^	124.58 (4)	O4—C4—C5	112.34 (15)
S1—Na—S1^ii^	121.70 (3)	O4—C4—C3	110.35 (14)
O5^i^—Na—S1^iii^	86.16 (5)	C5—C4—C3	112.70 (14)
O13^ii^—Na—S1^iii^	88.41 (4)	O4—C4—H4	106.7 (16)
O12^iii^—Na—S1^iii^	20.89 (4)	C5—C4—H4	105.9 (15)
O1—Na—S1^iii^	89.30 (4)	C3—C4—H4	108.5 (16)
O2—Na—S1^iii^	110.71 (4)	O5—C5—C4	111.02 (15)
O11—Na—S1^iii^	163.98 (4)	O5—C5—H5A	112.1 (17)
C1—Na—S1^iii^	111.76 (4)	C4—C5—H5A	108.1 (17)
S1—Na—S1^iii^	140.21 (3)	O5—C5—H5B	109 (2)
S1^ii^—Na—S1^iii^	91.60 (2)	C4—C5—H5B	110.9 (19)
O12—S1—O13	114.87 (9)	H5A—C5—H5B	106 (3)
O12—S1—O11	112.80 (8)	C1—O1—Na	104.51 (10)
O13—S1—O11	110.92 (8)	C1—O1—H1O	112 (3)
O12—S1—C1	105.74 (8)	Na—O1—H1O	114 (3)
O13—S1—C1	104.52 (8)	C2—O2—Na	112.86 (10)
O11—S1—C1	107.22 (7)	C2—O2—H2O	109 (2)
O12—S1—Na	142.43 (6)	Na—O2—H2O	108 (2)
O13—S1—Na	102.56 (6)	C3—O3—H3O	108 (2)
O11—S1—Na	45.97 (6)	C4—O4—H4O	111 (3)
C1—S1—Na	65.75 (6)	C5—O5—Na^vi^	134.32 (12)
O12—S1—Na^iv^	93.81 (6)	C5—O5—H5O	108 (3)
O13—S1—Na^iv^	30.79 (6)	Na^vi^—O5—H5O	118 (3)
			
O12—S1—O11—Na	−142.14 (7)	O12—S1—C1—Na	140.92 (7)
O13—S1—O11—Na	87.39 (8)	O13—S1—C1—Na	−97.46 (7)
C1—S1—O11—Na	−26.15 (9)	O11—S1—C1—Na	20.34 (7)
Na^iv^—S1—O11—Na	87.43 (9)	Na^iv^—S1—C1—Na	−125.05 (4)
Na^v^—S1—O11—Na	−174.14 (5)	Na^v^—S1—C1—Na	142.05 (6)
O13—S1—O12—Na^v^	64.12 (12)	O1—C1—C2—O2	−55.53 (17)
O11—S1—O12—Na^v^	−64.32 (11)	S1—C1—C2—O2	63.32 (15)
C1—S1—O12—Na^v^	178.80 (9)	Na—C1—C2—O2	−13.98 (11)
Na—S1—O12—Na^v^	−110.68 (9)	O1—C1—C2—C3	61.35 (16)
Na^iv^—S1—O12—Na^v^	87.47 (9)	S1—C1—C2—C3	−179.80 (11)
O12—S1—O13—Na^iv^	50.59 (13)	Na—C1—C2—C3	102.90 (12)
O11—S1—O13—Na^iv^	179.96 (9)	O2—C2—C3—O3	175.85 (13)
C1—S1—O13—Na^iv^	−64.81 (12)	C1—C2—C3—O3	55.39 (16)
Na—S1—O13—Na^iv^	−132.66 (9)	O2—C2—C3—C4	−60.69 (16)
Na^v^—S1—O13—Na^iv^	82.62 (10)	C1—C2—C3—C4	178.85 (14)
O12—S1—C1—O1	−176.87 (11)	O3—C3—C4—O4	58.72 (17)
O13—S1—C1—O1	−55.25 (13)	C2—C3—C4—O4	−62.77 (18)
O11—S1—C1—O1	62.54 (12)	O3—C3—C4—C5	−67.77 (18)
Na—S1—C1—O1	42.21 (9)	C2—C3—C4—C5	170.74 (14)
Na^iv^—S1—C1—O1	−82.85 (10)	O4—C4—C5—O5	66.8 (2)
Na^v^—S1—C1—O1	−175.75 (7)	C3—C4—C5—O5	−167.74 (14)
O12—S1—C1—C2	63.56 (13)	C2—C1—O1—Na	60.41 (13)
O13—S1—C1—C2	−174.82 (12)	S1—C1—O1—Na	−61.72 (10)
O11—S1—C1—C2	−57.02 (13)	C1—C2—O2—Na	19.43 (15)
Na—S1—C1—C2	−77.36 (11)	C3—C2—O2—Na	−101.16 (12)
Na^iv^—S1—C1—C2	157.59 (11)	C4—C5—O5—Na^vi^	−108.80 (17)
Na^v^—S1—C1—C2	64.69 (15)		

Symmetry codes: (i) *x*, *y*, *z*−1; (ii) *x*, *y*+1, *z*; (iii) *x*−1, *y*+1, *z*; (iv) *x*, *y*−1, *z*; (v) *x*+1, *y*−1, *z*; (vi) *x*, *y*, *z*+1.

### Hydrogen-bond geometry (Å, º)

**Table:**

*D*—H···*A*	*D*—H	H···*A*	*D*···*A*	*D*—H···*A*
C5—H5*B*···O11^vii^	0.97 (4)	2.48 (3)	3.394 (2)	157 (3)
O1—H1*O*···O11^viii^	0.74 (4)	2.00 (4)	2.6813 (19)	152 (4)
O2—H2*O*···O1^ix^	0.88 (4)	1.97 (4)	2.8311 (19)	164 (3)
O3—H3*O*···O4^viii^	0.87 (3)	1.79 (3)	2.664 (2)	173 (3)
O4—H4*O*···O13^vi^	0.86 (4)	2.11 (4)	2.936 (2)	162 (4)
O5—H5*O*···O3^ii^	0.80 (5)	1.99 (5)	2.782 (2)	166 (5)

Symmetry codes: (ii) *x*, *y*+1, *z*; (vi) *x*, *y*, *z*+1; (vii) *x*−1, *y*, *z*+1; (viii) *x*−1, *y*, *z*; (ix) *x*+1, *y*, *z*.
